# Enacting inclusive science: Culturally responsive higher education practices in science, technology, engineering, mathematics, and medicine (STEMM)

**DOI:** 10.1371/journal.pone.0293953

**Published:** 2024-01-17

**Authors:** Krystle P. Cobian, Sylvia Hurtado, Ana L. Romero, Justin A. Gutzwa

**Affiliations:** 1 Department of General Internal Medicine, David Geffen School of Medicine, University of California, Los Angeles, Los Angeles, California, United States of America; 2 Department of Education, School of Education and Information Studies, University of California, Los Angeles, Los Angeles, California, United States of America; 3 Department of Educational Administration, College of Education, Michigan State University, East Lansing, Michigan, United States of America; Instituto Tecnologico Autonomo de Mexico, MEXICO

## Abstract

Novel approaches in higher education are needed to reverse underrepresentation of racial/ethnic groups in science, technology, engineering, mathematics, and medicine (STEMM). Building on theoretical frameworks for practice in diverse learning environments, this study provides evidence for Inclusive Science as a conceptual model that reflects initiatives intended to diversify biomedical research training for undergraduates. Using multiple case study design and cross-case analysis, we analyzed data from 10 higher education sites that were awarded the Building Infrastructure Leading to Diversity (BUILD) grant funded by the National Institutes of Health (NIH). We identified the following dimensions of the Inclusive Science model: promoting participation of diverse researchers; introducing diversity innovations in science and research curriculum; improving campus climate for diversity; providing tangible institutional support; creating partnerships with diverse communities; and integrating students’ social identities with science identity. We illustrate each dimension of the model with examples of campus practices across BUILD sites. While many may doubt that science can be responsive to diversity, the interventions developed by these campuses illustrate how colleges and universities can actively engage in culturally responsive practices in STEMM undergraduate training that integrate trainees’ identities, knowledge of diverse communities, and create a greater awareness of the climate for diversity that affects student training and outcomes. Implications include culturally responsive strategies that many more higher education institutions can employ to support scientific career training for historically excluded groups.

## Introduction

A congressionally mandated report indicated the U.S. was at a crossroads in addressing diversity in science, technology, engineering, mathematics, and medicine (STEMM) degree attainment, pointing to a need to at least triple the number of degrees among the growing number of college graduates from underserved communities [1]. Ten years since that report, there has been virtually little change: African Americans remain underrepresented at all degree levels, whereas Latine/Hispanics and American Indians/Alaska Natives are underrepresented at all but the associate’s degree level [2]. Why has there been relatively little change in racial/ethnic representation among students receiving degrees in STEMM fields? Although student interest has increased among STEMM majors across undergraduate racial groups, opportunities and completion rates are not equal [3], and policies and practices allow forms of systemic exclusion to remain intact [4]. As a result, individuals are left to navigate academic environments they perceive as hostile to their underrepresented identities [5, 6]. However, the murder of George Floyd in 2020, rising racially-motivated hate crimes, and subsequent social protests sparked national conversations in scientific academic organizations [7, 8] in an effort to identify ways to dismantle structural racism and bias [9]. The NIH also took action to review and address structural racism, targeting processes, research and training programs, as well as its own organization [10]. College campuses that address equity and inclusion, with NIH support, continue to do so with renewed focus and efforts. However, novel approaches and practices are needed to increase and guide participation from historically excluded groups in STEMM.

The purpose of this study is to present the evidentiary basis and modification of a model of Inclusive Science developed at the early stages of a multi-campus NIH-funded initiative aimed at enhancing diversity in the biomedical science workforce. We set out to extend the framework in this study of campus strategies to enhance diversity in their approaches to training undergraduates in STEMM, focusing on the Building Infrastructure Leading to Diversity (BUILD) initiative that is part of the NIH-funded Diversity Program Consortium (DPC). BUILD institutions received funding to strengthen their training of biomedical researchers, focusing on proposed plans to address student, faculty, and institutional capacity development. Development of the initial model of Inclusive Science was largely based on campus proposals and descriptions of early implementation, and subsequently presented to grantees at an annual meeting [11]. As sites approach the end 10 years of funding, there have been several DPC-wide publications that provide evidence of the initiative’s outcomes [12, 13]. This study provides a theoretical contribution to STEMM education and training efforts, as well as practical guidance on how culturally responsive practice is enacted in key domains of undergraduate science training. While several of the components of Inclusive Science are evidence-informed and based on previously published social science research, the model envisions how culturally responsive, evidence-based practices can be enacted at a larger-scale organizational level to advance diversity in STEMM training. Many campuses may learn from institutional practices in STEMM when they see examples of how it is possible to design science training initiatives that are inclusive, culturally responsive, and address structural issues.

### The BUILD initiative

BUILD awards are linked grants issued to undergraduate institutions to implement and study innovative approaches to support students from diverse backgrounds in biomedical research, potentially helping them on the pathway to become future contributors to NIH-funded research [14]. Awardee sites partner with community college and research-intensive institutions, to broaden the pool of students participating in biomedical research training and maximize opportunities for faculty and staff development [15]. Each BUILD site has a shared structure of cores for administration, institutional development, student training, and research enrichment [15]. BUILD activities focused on students typically included: financial support, enrollment in redesigned STEMM curricula, diversity training, academic advising, mentoring, research training, and biomedical career development activities. BUILD sites created scholar, associate, or trainee programs for a core group of students who received the most intense exposure to such activities. At the same time, BUILD student initiatives reached beyond the traditional notion of intensive mentor training on a small cohort of students as each site sought to scale certain activities to reach a larger amount of students apart from the few admitted into their site’s scholar/trainee program [15]. While the 10 BUILD sites were guided by the parameters of the award, each site separately designed and proposed activities for their respective programs. Sites adapted several evidence-based STEMM education practices into their own context to work with underrepresented groups and reduce equity gaps. For example, sites focused efforts on revamping undergraduate biomedical curricula via active learning [16] and course-based undergraduate research experiences (CURES) [17]. Other sites adopted paradigms for biomedical training such as the One Health approach, which has been documented as a promising holistic multidisciplinary approach to science [18].

For this study, we use the term PEERS (persons excluded due to ethnicity and race) [19] to refer to individuals that NIH has designated as underrepresented in the biomedical, clinical, behavioral, and social sciences workforce, including individuals who identify as Black or African American, Latine or Hispanic, and American Indians or Alaska Natives, Native Hawaiians and other Pacific Islanders [20]. Campuses that include other targeted groups in their initiatives will be noted. The study focuses on multiple dimensions of a frame that reflect strategies developed to address underrepresentation in biomedical sciences across campuses in the NIH-funded BUILD initiative.

## Frameworks guiding inclusive science

Campuses can be diverse, but not inclusive in science education and training. Additionally, campuses can provide science training opportunities for excellent or exceptional students from historically excluded backgrounds but may not enact efforts to expand opportunities that could unlock the potential for several more individuals. For example, some college campuses reserve science training opportunities for the few “top students’’ as part of a deeply embedded notion of excellence in science. Inclusive excellence was a term introduced as a set of principles to indicate that diversity and excellence were not mutually exclusive concepts, and that all college students can learn at high levels [21]. Yet, many failed to question notions of excellence and commit to equity as part of inclusive excellence, requiring renewed national efforts [22]. In contrast with inclusive excellence, the term inclusive science connotes a “science for all” approach. Inclusive science is the term scholars used in initial studies of practices for students with disabilities as a way to make science learning more accessible [23], and to advance university faculty training to address androcentric and ethnocentric bias [24]. Inclusive science is tied to systemic change by questioning notions of who is considered “excellent” by current systems and standards in science, and enacting practices to support students historically excluded from science education and career training opportunities. Inclusive Science was introduced as a framework for describing BUILD activities, based on proposed culturally relevant approaches that are integrated into a variety of science training activities that signal institutional change, including the curriculum but also going beyond it to address many areas important to PEERs in STEMM [11]. This study extends and modifies this initial work to provide empirical examples of practices that illustrate Inclusive Science practices in NIH-sponsored initiatives.

### Inclusive science centers identity and culturally responsive practice

Key models and principles that center student identity in classroom and extracurricular practices inform the Inclusive Science model. Scholars have conceptualized culturally relevant, responsive, and sustaining pedagogy in K-12 education [25–27]. Collectively, this evolving body of literature points to the need for educators to incorporate culturally responsive practices to support student success. In contrast to deficit approaches to education and training, whereby PEERS are viewed as lacking skills, abilities, or resources and are thus less capable, culturally responsive approaches view PEERS’ social identities and cultures as an asset and means for education itself, rather than a barrier to overcome [27, 28].

The key elements of the theory of culturally responsive pedagogy [25] have been applied to urge the transformation of science departments and include creating learning environments “where students do not experience conflict between their lives as a science student and other parts of their identities,” and opportunities to think critically about the culture of science that benefits some students and may disadvantage others [[29] p. 3]. Although the latter study extends culturally responsive pedagogy to embrace broader transformation, it was limited to department-level curriculum change efforts in STEMM disciplines. In this study, we extend Inclusive Science to be a framework that expands culturally responsive approaches in education, focusing on institutional practices implemented by faculty and administrators to attract and retain students in biomedical fields at all levels of higher education. Inclusive Science also acknowledges the value of cultural connections to communities as a motivating factor among PEERs, particularly among first generation science students [30]. An additional element of culturally responsive practice recognizes the climate for racial/ethnic diversity as a factor in student transition, adjustment, and persistence in college and in STEMM. This is derived from much research on the experiences of PEERs in college, which posits student identities are at the center of faculty and staff practices designed to achieve student success [31]. Faculty and staff not only provide material for students to learn via science course curricula, but also provide socialization for careers, a sense of belonging, and validation of students’ current and developing identities. These interactions with institutional agents are shaped by the campus’ climate for diversity, which is reflected in predominantly white higher education institutions’ historical legacy of exclusion, compositional diversity, institutional infrastructure of support, psychological perceptions, and the quality of interactions on campus [31]. As part of culturally responsive practice, recognition of these identity and racial dynamics are important in the environment of underrepresentation that PEERS must negotiate in order to persist in science [32, 33].

## Methods

### Multiple case study design

The research team employed a multiple case study approach [34, 35], focusing on how different institutional contexts shaped the larger phenomenon of interest. For this study, we were interested in campus practices and strategies from the 10 BUILD sites that reflected Inclusive Science to refine the originally conceptualized model developed during early phases of sites’ BUILD implementation. Case study research is a primarily qualitative technique in which a case or multiple bounded cases are examined in their real-life context(s) [35]. Case studies are useful for description of context-specific implementation of programs and practices that reflect explanation, exploration, and replication of cases.

#### Case selection

The data include documents and interviews with administrators, faculty, staff, and student focus groups at the 10 BUILD sites. In 2014, 10 NIH BUILD multi-year awards were issued to undergraduate institutions across the country. Eligibility for competitive project awards included having less than $7.5 million in total NIH research funding, and at least 25 percent Pell Grant recipients. The sites are composed of two historically Black colleges and universities (HBCUs), five Hispanic-serving institutions (HSIs), one of which is also an Asian American/Native American/Pacific Islander-serving institution, and the rest of the campuses targeted outreach to special populations that are underrepresented in biomedical disciplines. Each NIH-funded site employed different innovative approaches to develop capacity for meeting program goals, including research skill building, training (for students and faculty), and infrastructure development [36].

#### Site visits

From 2017–2018, the research team visited each BUILD site to conduct interviews, focus groups, and observations of the BUILD initiatives. Approved by the institutional review board at UCLA (IRB#15–002023), we obtained written consent from all participants in the study prior to conducting interviews. The team interviewed BUILD program leaders, program coordinators, faculty training participants, student participants, and senior leaders at the institution to understand the BUILD program at the local site and implementation strategies. Working with program principal investigators (PIs) or designated staff, the researchers identified and recruited over 500 staff and faculty participants in BUILD for individual interviews and focus group participants (see technical report, [37]. Interviews and focus groups ranged from 45 to 90 minutes each and were recorded, transcribed, and uploaded to Dedoose, a qualitative data analysis software package. At the conclusion of each site visit, the site visit team conducted a debrief meeting with BUILD program leadership to offer insights and clarify observations.

#### Coding and analyses

The research team developed a codebook to account for institutional-wide, program-specific, faculty-specific, and student-specific levels of activities. The codebook deductively examined how existing literature on diversity in the biomedical sciences and the DPC “Hallmarks of Success” were present in the data. To understand BUILD implementation, the team also generated codes inductively to capture the exploratory nature of aspects of BUILD implementation that did not fit within the codes generated to align with the DPC Hallmarks of Success [38]. Coders also employed descriptive coding [39, 40] to summarize and organize the primary topic of excerpts. To ensure inter-rater reliability, or extent of agreement among data collectors with respect to interpretation of the phenomenon of interest [41], coding team members participated in an inter-rater reliability test on Dedoose using Cohen’s kappa statistic [42]. An intercoder reliability score of .96 across all ratings was reached based upon a pooled kappa estimate [43]. The research team created debrief reports after each site visit, which were shared with BUILD site PIs and local site leaders (see technical report, [37]). Using the debrief reports, transcripts, and codes, researchers wrote case narrative reports for each site in order to summarize themes within each site’s context [34]. Internal case narrative reports contained summaries and general impressions of each case as a first step in analysis.

#### Cross-case analysis

While the research team generated data for the larger project, only the co-authors were involved in the final stages of analysis and writing for this specific study. Additionally, while a prior group of co-authors conceptualized and published the first version of the Inclusive Science model [11], only one co-author from the previous group was involved in the present analysis to systematically obtain evidence of the previously theorized model. We employed cross-case analysis to identify similarities and differences among the sites that inform the phenomenon of interest: dimensions of Inclusive Science [34]. We deductively sought evidence of each dimension of the original Inclusive Science model, while also inductively analyzed the case study data to generate modifications to the model. To do this, the co-authors used debrief reports; case narrative reports; BUILD site websites; a special issue featuring articles summarizing each BUILD site’s intended goals and initiatives [11], as well as relevant coded excerpts and raw transcripts in order to gain a holistic and nuanced understanding of patterns and themes. The co-authors ran data queries using Dedoose to pull specific codes related to the initial dimensions of Inclusive Science. To inductively analyze the data, the team read case narrative reports for each site and located additional codes and transcripts seeking evidence against the model, as well as evidence of additional practices not yet captured by the original conceptualization of the Inclusive Science model. To organize the data and analytical process, the co-authors created matrices to visualize the extent to which each BUILD site exhibited evidence of engaging in efforts related to the dimensions. The use of matrices allowed researchers to make contrasts and comparisons between institutions [39] and determine when the co-authors reached saturation for a particular assertion.

To further organize the large amount of case study data, the co-authors employed focused coding [44], and wrote analytic memos to make sense of the data and develop assertions for each Inclusive Science dimension. We met weekly during analysis to discuss findings and agreed upon a revised model and corresponding subthemes for each dimension of the model.

#### Limitations

Limitations to this study include a focus on sites that were selected to receive funding from NIH as part of the BUILD initiative. Many campuses across the country employ interventions, and few will ever receive a substantial level of federal funding for comprehensive program initiatives lasting up to 10 years. However, case study research is not intended to be broadly generalizable; instead, it is focused on replications of cases, with each providing contextual insights into how these campuses utilized the grant funding and responded to the parameters of the award. Through explanation of each dimension of Inclusive Science that we identified, we offer a variety of examples so that other campuses might identify strategies that align with their own institutional context and constraints to advance Inclusive Science and PEER degree completion in STEMM.

The current study focused on the early years of implementation; therefore, a second limitation is that student outcomes are not the primary focus of the current analysis. While an analysis connecting Inclusive Science practices to outcomes is beyond the scope of this current study, future research will be able to make connections between strategies employed by sites and tangible outcomes such as biomedical graduation rates, persistence into biomedical graduate study, and measures of psychosocial outcomes such as science identity and self-efficacy as a result of BUILD interventions. Despite BUILD being in the early implementation and institutionalization phase of their respective programs, each BUILD site as well as the central evaluating body, the Coordination and Evaluation Center (CEC), has published evidence of outcomes [12, 13, 45]. As of fall 2022, BUILD sites have collectively supported over 2,473 undergraduate students—a majority of whom identify as individuals from groups considered underrepresented in STEMM by NIH—via participation in each site’s most intensive BUILD scholar and associate program activities [46]. Early findings indicate that participation in the BUILD scholar and associate program is associated with higher levels of reported science identity [47], and self-efficacy as a scientific researcher [48]. Additionally, BUILD program elements also expanded their programmatic efforts to support additional students on each campus via undergraduate research opportunities, curriculum changes, and professional development initiatives. Among student participants in the evaluation, the CEC has collected survey data from over 30,000 unique undergraduate student respondents at all BUILD sites [49]. The CEC works in collaboration with local BUILD evaluation teams to track participation in such activities in order to capture the reach of the BUILD initiative [50]. Local evaluations of BUILD have found a host of positive outcomes from BUILD-initiated department-level and institutional-level interventions aimed to increase quality active learning curricula in introductory science courses [51, 52]. The importance of mentorship for supporting biomedical research career development [47], whether from peers [53], professional staff, or faculty [54] also has also been a persistent finding from multiple BUILD sites. Out of the DPC evaluation, the National Research Mentoring Network has developed measures of biomedical mentors’ capacity to be culturally aware in mentor-mentee relationships [55]. Some BUILD sites developed successful models for providing mentoring in biomedical research to students from historically excluded groups. Due to interest in findings from this historic initiative, BUILD sites are developing plans to continue to evaluate the impact of the program beyond the last year of funding for its implementation.

Lastly, while not the focus of this study, another main aim of BUILD is to evaluate its efforts to disseminate findings that can inform the STEMM education community. For context, the number of first-year undergraduates alone who are enrolled in the evaluation of the project (includes students who participated in BUILD activities versus students who were not exposed to BUILD) include 32,963 first-year students; with 27% from families with an income < $30,000 annually and 25% reporting that they were first generation college students [49]. Throughout the findings for this study, we clarify whether participants reported program outcomes of Inclusive Science practices that they employed, or whether the co-authors identified outcomes based on triangulating data from multiple participants.

## Findings: Dimensions of inclusive science

**Fig 1:**
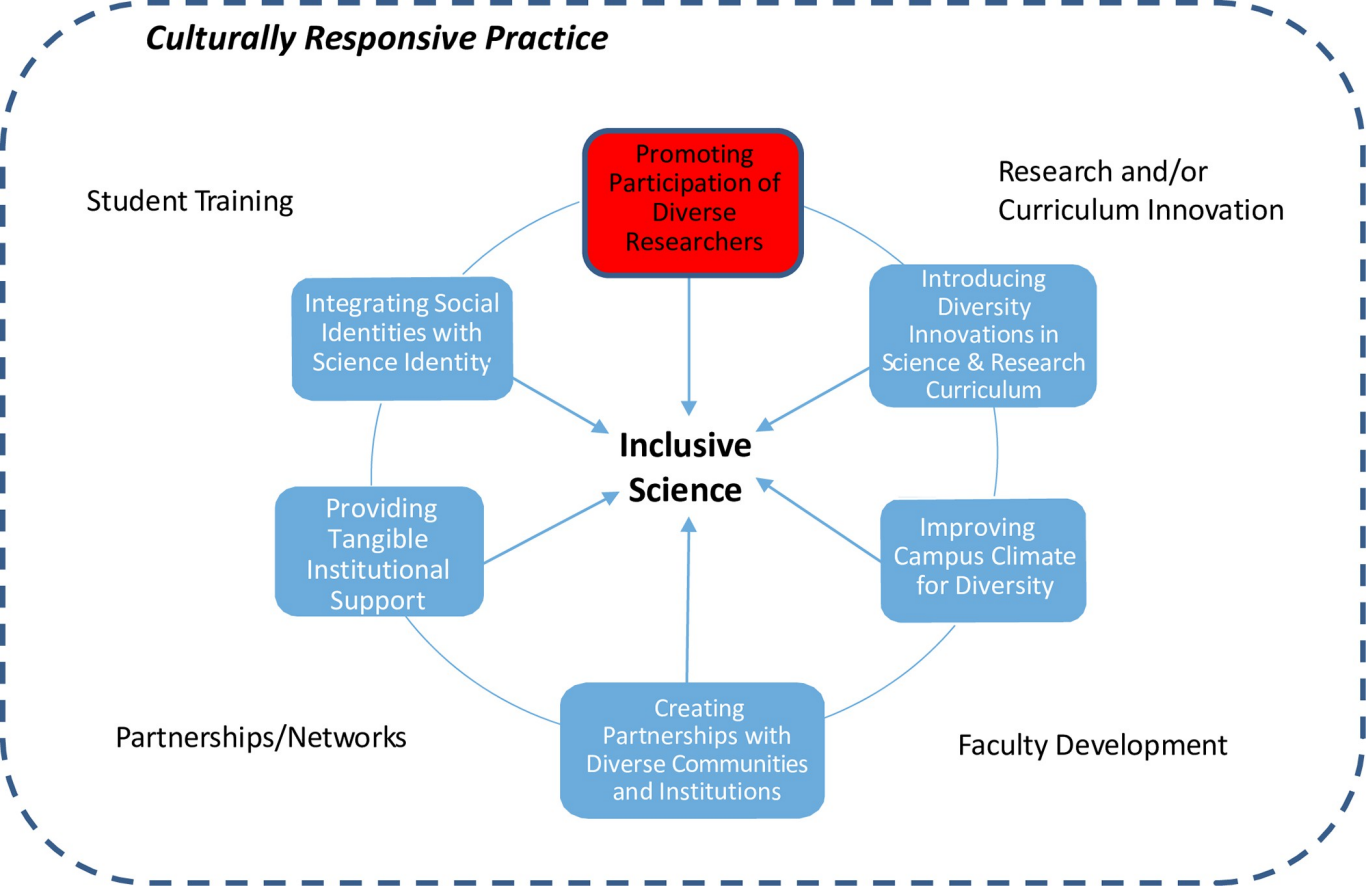
Enhanced inclusive science model. Adapted from [11].

The Inclusive Science model resulted from case study analyses, with two significant modifications to the original model [Fig 1]. Each dimension is further elaborated with key themes identified with evidence from institutional practices. First, culturally responsive practice was originally a separate dimension within the Inclusive Science model [11], but it became evident from BUILD cases that all dimensions of Inclusive Science embed cultural responsiveness in practice. Second, we noted significant investment in what we call tangible forms of support for student success—an area missing from the original model but central to the kinds of structural support necessary for building inclusive diversity initiatives [31]. The model indicates that Inclusive Science is central to the main areas of functional activity in research/curriculum content innovation, faculty development (e.g., teaching and mentoring), partnerships for recruitment and retention, and student research training. It should be noted that the Inclusive Science dimensions are interrelated, and campuses often employed more than one dimension within comprehensive BUILD initiatives. The next sections provide examples of practices in each of the dimensions of Inclusive Science.

### Dimension: Promoting participation of diverse researchers

A central goal of an Inclusive Science approach is to develop a talented pool of researchers from diverse backgrounds and reduce disparities in representation across biomedical disciplines and career stages. In this study, campuses extended initiatives to PEER groups and other excluded social identity categories, opening multiple pathways for students. At several of the sites, administrators expressed their belief in the value that diverse researchers bring to biomedicine. For example, an administrator at a San Francisco State University (SFSU) partner institution, the University of California, San Francisco, makes clear the link between the lack of individuals with diverse experiences and perspectives and the gaps in scientific knowledge:

As an institution, we’re not just committed to training minority trainees. We’re committed to making a difference in the community…for me the biggest distinction that we make, and I think is wrong, is we separate disparity from diversity. I don’t think we can. To have a more diverse researcher, the research has to address disparities. To have better, more health equity, that needs a more diverse researcher. It’s a spiraling conversation.

This point highlights that it is essential to attract diverse researchers not simply to find different perspectives, but to address societal issues in marginalized communities (e.g., inequitable access to healthcare) through scientific discovery.

BUILD sites worked to promote the participation of researchers traditionally underrepresented in STEMM by 1) increasing participation along the entire undergraduate education pathway, 2) removing barriers to recruitment and selection of scientific talent, and 3) expanding definitions of target populations to train in biomedical research.

#### Increasing participation along the undergraduate pathway

BUILD sites actively increased the number of potential biomedical participants along the undergraduate career pathway by allowing undergraduate students different entry points into biomedical research as a career option. While four of the 10 BUILD sites had programming for first-year students, the other six BUILD sites created program structures to allow students to apply for BUILD-related scholar programs and activities during sophomore, junior, or senior year, in addition to providing opportunities for community college students to apply to BUILD. The flexibility of the programs allowed students to enter biomedical career training at multiple points during their time in college. BUILD administrators explained the importance of providing multiple opportunities to expose students to biomedical career training in order to broaden perceived career possibilities and allow time for students to develop biomedical interests. For example, Xavier University of Louisiana (XULA) focused on coordinating all programs that result in a developmental sequence of research experiences throughout the college years. XULA also developed a post-baccalaureate program for students who missed research opportunities during their undergraduate years and/or lack research experience and preparation needed for graduate school. Thus, XULA’s program structure provided additional entry points into biomedical research experience and graduate study.

#### Removing barriers to recruitment and selection of scientific talent

BUILD sites aimed to remove barriers to recruitment and selection of scientific talent through simplifying application processes, especially after realizing that requirements may have prevented some students from completing the application and as a result, never begin their pathway into a biomedical career. For example, the University of Detroit Mercy (UDM) changed their application process to allow students to come in for drop-in interviews for the BUILD program. While this was only a slight change to the process, one administrator noted, “I’m really surprised at what an impact that made in terms of increasing our pool of prospective students. [Introducing flexibility] removed a lot of the obstacles that I didn’t even know were obstacles.” Sites also removed barriers by broadening their application processes to include social science fields that overlapped with biomedical research and opening the application to individuals who initially had healthcare career aspirations rather than biomedical research career goals. These changes were based on a belief that this could be impactful for first generation college students, who are often encouraged to go into careers that seem more familiar (e.g., healthcare rather than biomedical research). Xavier University Louisiana (XULA) specifically expanded their application to include students who indicated interests in medical or pharmacy school. BUILD administrators believed that if they could get more students involved in undergraduate biomedical research training, they could increase students’ interests, self-efficacy, and intention to pursue a biomedical research career in addition to, or instead of, initial career goals aimed at healthcare careers. As part of recruitment and attracting a larger pool of potential applicants, other sites also provided ways for trainees to see science as a way to “give back” to their communities and as a way to encourage and connect science to larger societal goals.

#### Expanding definitions of target populations to train in science research

NIH recently changed their definition of underrepresented groups (URGs) in order to more accurately address the issues of diversity and inclusion in STEMM. Portland State University (PSU) expanded their definition of who they consider “underrepresented” by including foster youth. In addition, PSU partnered with community colleges and four-year institutions in the Pacific Rim (e.g., Guam, American Samoa) and Alaska in order to target efforts toward other underserved groups such as Pacific Islander students. Providing opportunities and comprehensive support to individuals who may not typically have access to resources to propel success in biomedical science fields is critical for increasing participation of researchers from diverse backgrounds. However, while diverse groups produce higher rates of novel scientific research, their novel contributions are often devalued in the scientific workforce [56], which leads to the next dimension of expanding research and curricula arenas to better reflect PEER communities and research issues.

### Dimension: Introducing diversity innovations in science and research curriculum

Integration of diversity in scientific training, teaching, and inquiry is pivotal to developing an inclusive approach to science. Inclusive Science acknowledges the need to change the curriculum and approaches to research. The curriculum aims to teach students the skills for conducting research and integrate interdisciplinary approaches to teaching and learning. Training biomedical researchers to conduct interdisciplinary research allows for a more collaborative, holistic approach to addressing health inequities and gives way for new areas of research to emerge. Culturally responsive innovations in science and research also grapples with historical mistrust of science and rebuilding trust with marginalized communities that have been adversely impacted by research.

To achieve these aims, BUILD institutions developed courses, modified teaching and pedagogy training, developed new lines of research, and engaged the needs of diverse communities. Institutions aimed to prepare students to engage in research that is both personally meaningful and “moves the science forward.” This dimension includes 1) curriculum reform and research training, 2) interdisciplinary approaches to teaching in science, 3) new research on diverse communities, and 4) acknowledging histories of distrust in science research.

#### Curriculum reform and research training

Within curriculum reform and research training, course (re)development was the most prevalent approach BUILD sites used to attract students. BUILD institutions developed courses for undergraduate students with the intent to develop their researcher self-efficacy and science identity. Courses primarily focused on training students on the mechanics of research and/or introducing them to interdisciplinary approaches to examining health disparities. Some BUILD programs required these courses for BUILD students and used them to prepare students who may not otherwise participate in research. Courses aimed to engage students who may otherwise be left out of research experiences and make students better prepared to undertake research. Many courses taught how to develop a research proposal, methodology, and theory, while others engaged students in practical application of research skills. At California State University, Long Beach (CSULB), students had the option to enroll in introductory and advanced research methods courses as well as classes to prepare them for other stages of the research process, such as grant writing. While many universities offer a summer bridge program, Morgan State University (MSU), for example, was unique in offering a summer training institute based on an entrepreneurial model whereby students are trained to design their own research and obtain funding, rather than the traditional apprentice model of research. During the eight-week summer program, prospective BUILD students learned “how to do health research” and prepare proposals.

By having students develop proposals and implement their project, faculty and administrators at MSU sought to develop students’ researcher self-efficacy. A program director explained that students can go on to courses that “provide them with more theory and more methodological background needed to complete the research” with the additional goal of preparing them for graduate studies. Developing students’ foundational research skills was critical across BUILD programs.

Notably, research courses were also utilized to develop students’ science identity and self-efficacy as researchers. Enrolling in laboratory courses enabled students to apply the skills they learned in previous coursework. By learning and applying research methods, BUILD institutions simultaneously aimed to teach students how to do research as well as “getting them to start to think like a scientist” (program faculty leader at Wayne State University, a partner institution of UDM), “instead of consuming knowledge” the courses encourage them to “start thinking about how they can learn to create knowledge.”

While this approach may be similar to current practice in research training, the intentional inclusion of underrepresented populations in these classes, and the added effort to structure training into programs and curriculum removes the guesswork for PEERS to enter biomedical training experiences. STEM BUILD at University of Maryland, Baltimore County (UMBC) includes community college students in their summer training course, thus reaching populations who typically do not have high rates of participation in undergraduate research. These efforts show that Inclusive Science focuses on preparation as well as enrollment of PEERs.

#### Interdisciplinary approaches to teaching in science

BUILD institutions were invested in an interdisciplinary approach to teaching and training students on health disparities topics. Faculty and administrators emphasized the necessity of demonstrating how biomedical, social, and behavioral sciences, and the humanities are important in examining health-related topics. A CSULB program administrator shared:

I think more and more research is understanding you can’t just hang out in your little silo and move things forward; you have to work with other people. I think it’s really important for them to get a taste of that during their training so that they understand how more minds coming together and bringing in diverse ideas and thoughts from different, not just diverse people, but diverse disciplines, how that really enriches the research process and how it helps us to better understand what we’re trying to answer. It just brings a lot more expertise and resources to the table… that’s something that we really want to instill in [students].

Engaging researchers from different disciplines is viewed as necessary for the future of research and to address health disparities. Having students see the intersections of these disciplines as highly important in moving research forward guided curriculum development and training of biomedical students. Faculty developed courses across STEMM, social sciences, and humanities disciplines that focused on health-related topics. Several faculty used course-redesign grants provided by BUILD to develop new classes.

Additionally, several BUILD programs developed partnerships with departments in the social and behavioral sciences intended to establish interdisciplinary courses. For example, a faculty member at California State University, Northridge (CSUN) used a course redesign grant to develop a course on Asian Americans and health-related issues that differ by race/ethnicity. Additional examples of courses at other institutions included a race, science, and technology course at UDM, a collaboration between biology and dance at University of Alaska, Fairbanks (UAF), and a philosophy of ethics course at XULA. The University of Texas at El Paso (UTEP) developed several courses such as a sociology class under the Course-Based Undergraduate Research Experience (CURE) program, a health psychology course, and a mobile health technologies course in engineering. BUILD support gave way for faculty to develop courses that show students the relationship between a variety of disciplines and health-related issues.

A few programs trained students by exposing them to interdisciplinary research through presentations or mentoring experiences. Institutions used these approaches to bring faculty and researchers in disciplines outside of STEMM to show students how they can draw on other disciplines such as social sciences to inform their research and expand their understanding of a problem. Students were also exposed to interdisciplinary research by working with faculty in departments different from their own. A faculty member at UTEP described exposing their engineering student mentee to research in nursing, and at MSU a faculty member in psychology said that students in biology or engineering might seek them out because of the research they do. At SFSU, lab rotations were used to introduce students to interdisciplinary research.

#### Research on diverse communities

BUILD institutions identified and developed courses that connected health-related research to issues of diversity and social justice. For example, UAF planned to require all undergraduate students to take an Alaska Native themed course, CSULB required BUILD students to take a health disparities course, and CSUN developed a race and technology class. These courses were intended to teach students about the connections between diversity and social justice and their relationship to health issues.

Institutions also focused on changing how they conduct research and departments became more responsive to students’ experiences and community needs. BUILD institutions were interested in tapping into students’ experiences and getting them to connect research skills to topics and issues of interest. UAF employed their locally developed One Health paradigm, whereby the relationship between human health, animal health, and the health of the environment are inextricably linked, as a way of connecting research with what is happening in students’ local communities [57]. A program director at UAF shared how this culturally responsive approach to looking at science resonated with students:

We were getting zero traction in being able to say, ‘Come to the university and learn how to pipette. Come to the university and learn about viruses. Come to the university and learn about molecular DNA work.’ But if we said ‘One Health,’ that’s what we’re here to teach you and work with you on…a concept that you’re already familiar with… That you live in the ecosystem and the health of the ecosystem where you hunt, fish, and live depends on your understanding of it… your interaction with that ecosystem at a medical level. They just went, ‘Yeah, I can do that because that’s important in my community. It’s important for my rural lifestyle and it’s important to my family and it’s important to my region. For me to understand the health of the fish, the health of the birds, the health of the ecosystem, climate change, virus problems.’

A UAF faculty member shared that it was important to make a “connection between the material that they’re learning and what’s happening in their world at home.” Similar sentiments were shared at other BUILD institutions, which sought to build research rooted in community needs. For example, a faculty member at CSUN shared the following story about a BUILD undergraduate participant and mentee who found meaning in connecting her community interests to research:

[BUILD student] realized down the line after doing some work in non-profit and community organizations that she wanted to go back and get her [bachelor’s degree]… [She] talked about the non-profit she created, which is amazing, but then when it links to academic work and talking about research, I think there was this thought about what research looks like. People have lab coats, beakers, smoke going off. My work is in implementation science and bridging and making academic community partnerships and linking her to a partner that in [local] county… When she saw, “This is research. We’re going out, we’re creating workshop materials on different mental health awareness topics all for the goal of connecting the Latino/Latina community to [county] health services” … now she’s working on this poster for [research symposium] where she’s taking the first author to lead it and realizing, “This is research. This is what this is about.” I think she needed to see the connections between what she was passionate about, and there’s also an area of research that’s big on community-based participatory research and bridging the science to practice gap.

Here, the faculty mentor’s culturally responsive practice of offering opportunities so that students can see how their interests can connect to biomedical research and benefit their local communities was critical to maintaining this Latina student’s interest in a biomedical career pathway.

#### Acknowledging histories and rebuilding trust in science research

Systems of education and health-related research have inflicted harm to marginalized populations and led to distrust of higher education by communities. The use of Black and Indigenous communities for medical research [58], the Tuskegee experiment, and the use of HeLa cells without proper consent from Henrietta Lacks, a Black woman, and her family, are all examples of health-related research that has caused harm and fueled mistrust. Inclusive Science requires a reckoning with historical harm and acknowledging that it will take considerable time and effort to rebuild trust with communities. The faculty lead at UAF described the importance of acknowledging this harm and repairing relationships between minoritized communities and the field of science:

Western education has not been a positive thing, but a very negative influence. And we took children away and forced them to learn English at a period that was very critical to the development of their culture…and so they lost a lot of their history and when they came back, after years at these boarding schools, they didn’t speak their native language, they couldn’t talk to their parents or their grandparents. They didn’t have the skills they needed. They didn’t need it in the Western culture; they needed it in their own culture. So when we go into the community and say, ‘Hey, we want to give scholarships.’ They see it as, you’re going to take [the children] away, they’re never coming back. And we’ve lost this tremendous [asset], our future, our resources, our [community’s] future.

Acknowledging that institutions of higher education adversely impacted the existence of Indigenous culture and language and the ability for communities to pass them down to future generations is a culturally responsive approach that seeks to be honest about past harms continuing to shape relationships between the “ivory tower of academia” and Indigenous communities. Building trust became foundational to collaborating and serving many of the Native and rural communities of Alaska. A workshop facilitator noted that “the issue of trust is a really, really big one in communities, and it takes time to develop that.” They noted that this could not be done overnight, and researchers needed to spend time cultivating those relationships, working with American Indian studies faculty. They recognize that anyone interested in doing research in the community must account for time to cultivate a trusting relationship with the community since “it can take just that first year to really have the dialogues you need to have.”

Inclusive Science requires that institutions not only demonstrate the relevance and benefits of research to the community but do so in collaboration with community members and prioritize their most pressing concerns. Incorporating reciprocity in research is a component of restoring relationships with marginalized communities. Not only is research done with a sense of purpose, but there are also intentional efforts to bring students and their findings back to the community.

### Dimension: Improving campus climate for diversity

Campus climate, originally defined as the current perceptions, attitudes, and expectations of members of an institution [59], has evolved to enhance our understanding of how and why views of the campus environment differ among historically marginalized groups. Now, campus climate not only focuses on psychological perceptions of the climate, but is also linked with changes in compositional diversity of a campus, behaviors based on interactions within and outside of class, and the socio-historical legacies of exclusion at the institution [31]. Campus climate, and its impact on students, is made explicit via assessment of students, staff, and faculty experiences [31]. While advancing an inclusive approach to science involves improving the climate in classrooms, labs, and interactions on campus for everyone involved in biomedical research, addressing the climate for undergraduate students in particular is critical. Research demonstrates the negative association between hostile campus climates and retention of PEERs in STEMM fields [32, 33, 60]. We found evidence of BUILD sites employing various training models aimed at providing faculty (and at times, graduate and undergraduate students) with awareness and skill-building to improve the campus climate for biomedical trainees from underrepresented groups. Most sites focused on improving the climate aimed to create affirming environments to increase psychological sense of belonging; however, some sites also sought to improve campus climate through more explicit focus on providing awareness and skill-building about socio-historical exclusion within science fields, higher education, and society.

#### Fostering affirming environments to increase psychological sense of belonging

We found evidence of faculty and staff learning how to better support students and create a psychological environment of support. Almost all sites offered some form of faculty mentor training (e.g., the National Research Mentoring Network [NRMN]’s Entering Mentoring or Culturally Aware Mentoring [CAM] training) with the goal of improving faculty capacity to mentor and train students in biomedical fields [61]. Faculty participants of these training sessions shared how the training helped improve their confidence in their ability to create a positive and affirming environment for their mentees and students in their classes. A few sites included “homework” or suggestions of practices that faculty could apply to improve the climate in their labs and classrooms. Some faculty expressed that they felt empowered with a larger toolkit to foster growth in students’ self-efficacy and science identity.

Sites also incorporated programs and events to build community among students and increase their sense of belonging to campus and the field of biomedicine. For example, UAF, UTEP, and PSU planned welcome events, socials, and mixers throughout the year to build community among BUILD-affiliated students, staff, and faculty. Several sites implemented learning communities and/or centers where students could socialize, study, and have a physical space where they belonged on campus.

In addition to creating physical environments & events to foster belonging, BUILD program administrators and faculty at many BUILD sites aimed to mitigate deficit-framed discussions about PEERs. An example of a deficit framing would be constantly reminding students that they lack math or science proficiency or are already at a disadvantage due to a lack of financial resources or lack of knowledge of the biomedical career pathway. To counter this, UTEP employed the strategy of utilizing an asset-based framework for their program [62] in which they reminded students about the assets they bring to science. Indeed, short-term interventions to remind minoritized students about the assets they bring to higher education have the potential to improve their overall capacity to thrive [63]. UTEP aimed to affirm their students’ potential and sense of belonging and is also in alignment with the university’s larger Student Success Initiative. Regarding ways that this approach gets put into practice with students, a senior administrator at UTEP shared:

Most colleges and universities…Their students come in and it’s just a set of problems and deficiencies that need to be overcome. That’s not our view… We’d ask [the students], "Well what do you think your assets are?" "Tell us about them?" And they looked at us, like… What are you talking about? Because no one through the education system in the schools really talked to them about that…we would toss out a suggestion, for example, "Well how many of you are bilingual or multilingual?" Everybody raises their hand and we say, well, don’t you think that, that’s an asset? And they looked at us and said, "Ah, that’s no big deal. Everybody speaks Spanish!" But no, it is a big deal…then we started talking about, well, where do we live? Well, we live in El Paso. Where is El Paso? It’s on the border. Well, maybe that’s an asset. You’re living in a very international community…[students] come to us with a bit of an edge and a bit of an advantage because of who they are and their life experiences. Now we’re going to help them hone those skills and those abilities so that they’re super-competitive. So they do great, they do well in the classroom and succeed academically but then they have the edge because they’re professionally developed. So we’re getting all faculty and in fact, including staff, engaged in this sort of professional development.

This asset-based approach employed at UTEP is also echoed by the faculty who support the BUILD program at UTEP. In an interview with two science faculty members and BUILD administrators, both faculty were adamant in sharing how talented their biomedical students were. One faculty member shared:

[The BUILD scholars] come from an underrepresented, probably poor background in most cases, but in my opinion, the students coming from those backgrounds are uniquely motivated compared to someone from a more affluent background. These students are just as capable as anybody else, so I don’t see it as a disadvantage at all to be honest. They’re bilingual. They’ve got a lot of assets they bring with them and it’s not a disadvantage, not one bit…I often hear that presented as a challenge, but it’s not a challenge. It doesn’t matter where anybody comes from, it matters where they go… I think that we find in a lot of our programs that the students are just as capable as anybody else and in many cases more motivated to succeed given where they come from.

Faculty and staff at UTEP and at other BUILD sites who expressed these attitudes toward their students often shared how these attitudes translated into students feeling like they not only belonged but could thrive and meaningfully contribute to biomedical research.

#### Developing awareness and skills to address systemic inequity

A few sites had components of their faculty training curriculum that more explicitly provided context about historical exclusion and systemic inequity. Some campuses introduced curricula from social science fields into faculty training or structured weekly discussions with faculty. These training sessions were critical for exposing STEMM faculty to important research and theories that impact PEERs, particularly because STEMM faculty members shared that it was the only space where they had an opportunity to learn about such topics. Faculty mentor training at a few sites incorporated the concept of microaggressions—brief, common exchanges that send denigrating messages to people of color or individuals from a non-dominant group [64–66]. SF State not only focused on providing faculty with opportunities to address structural exclusion in STEMM through their Faculty Agents of Change program, but also provided training on the psychological concept of stereotype threat (i.e., fear of conforming to stereotypes about one’s social group) to the students in the BUILD program. Their goal was to acknowledge the realities of structural inequity on campus, and to equip students with skills to empower them to navigate their educational and career training. This unique approach of equipping students with knowledge of socio-contextual factors that may influence their experiences in biomedical training appears to protect PEERs intellectual performance and safety from stereotype-based evaluative concerns [67].

One of the most intentional strategies for addressing structural exclusion came from CSUN. Through intentional employment of Critical Race Theory into its program implementation, CSUN assembled a CRT advisory board, who, along with other collaborators, developed and implemented a CRT training model so that biomedical faculty could improve their understanding of race and racism in biomedicine. One of the CRT advisory board members explains their progress and goals of the training:

We’re getting new people. In fact, we’re actually training administrators and staff this summer using a similar curriculum… .with similar issues about power and microaggressions, but pitched at administrators and staff instead of faculty…We’re seeing what people need and what people want from us, and the skills that they want to gain…[We say] we want you to be able to understand power dynamics, and understand that power is infused in everyday situations in what we do with science and it impacts people differently… That’s our end goal, we’re getting there bit by bit.

Some of the faculty at the BUILD sites at minority serving institutions (MSIs) became convinced that mentor training was beneficial for improving their campus climate. For example, one faculty member and BUILD program director at XULA (an HBCU) shared:

I have been a faculty member at Xavier. This is my 22nd year, and I’ve had 87 minority students as my research advisees in the lab over this time, but there are things that I’ve learned through the mentor training in the past few years that I never knew. One was, for example, the stereotypes, you know, the implicit bias. I was never really aware of it…I’ve learned these skills at the mentor training after all these years, I see that I’ve become a much better mentor to my mentees. The mentor training, I think, is a very important aspect of the program.

This faculty member clearly experienced the impact of training they received to create affirming environments, even at an institution with a historic legacy of providing an affirming environment to Black students. Faculty and staff spoke of the need to recognize campus climate and to gain additional skills to actively create positive environments and mitigate systemic challenges PEERs face. As one program administrator at a BUILD site shared, regarding the impact of developing the knowledge and skills to address exclusion, “[it] not only gives them knowledge of, ‘This is a stereotype’… But how to impact [their] trajectory in the sciences…for the faculty, what are ways they can change their classroom environment so that would be affirming for students so that they would experience stereotypes less.”

### New dimension: Providing tangible institutional support

The Inclusive Science model involves an emergent dimension that resulted from this study: Understanding students’ needs and meeting these needs through tangible student-centered services such as financial support, academic enrichment, tutoring, advising, and physical spaces for learning and identity development. Support can include other resources to bolster PEERs’ educational endowments, financial resources, and science socialization [62] that are not in other dimensions of Inclusive Science. Tangible forms of support demonstrate institutional investments to remove obstacles students may have during their undergraduate training and provide material resources as these students navigate their disciplines.

### Physical spaces: Biomedical classrooms, research spaces, and centers

Several campuses invested in classrooms, research spaces, and centers as places to provide students and faculty support and spaces to gather for research and community-building efforts. Centers were used as gathering spaces to build community and support systems, advance collaborative research, and share resources across students, staff, and faculty. Establishment of these physical spaces also symbolized institutional commitment to BUILD initiatives. Research centers, in particular, offered a place to continue collaborative research among students and faculty, and expanded on existing initiatives.

Building community and establishing a support network is critical to the success of underrepresented student populations and centers are critical in meeting this need. They give a place for BUILD students to come together and receive social, academic, and professional support. A director at UAF observed that students came to their center to “hang out” and connect with other students. At MSU, a shared space meant that the student research center led by a student organization provided a place to meet and form a sense of community in STEMM. For some of the institutions, such as PSU, the spaces gave students a place to come together to study and help one another. For other institutions, centers were places in which staff and faculty were made available to support students academically and professionally. At CSULB, the principal investigator shared that their BUILD center allowed for a “physical presence on campus” that centralized meetings with BUILD staff, faculty, space to complete various types of academic and co-curricular work, and hosted BUILD learning communities. Before the pandemic, the presence of such a space gave way for students to gather and connect with staff and faculty. Additionally, it provided a dedicated space to academic support. Similarly, at XULA, BUILD funds supported the addition of a center in physics to the existing resource centers dedicated to math, biology, and chemistry in the Student Academic Success Office. Given the multiple functions of physical spaces, centers can be used as places that anchor student and faculty support, and firmly establish the presence of BUILD programs.

#### Services to mitigate inequity

BUILD institutions also invested in staff and services to meet students’ needs to help them continue their biomedical research training. These services included 1) financial support, 2) academic support, and 3) career development. Helping students achieve their full potential meant identifying resources that remove barriers to participation in biomedical training activities and provide resources that helped students develop skills for future steps such as applying to graduate school.

Financial support is a significant aspect of BUILD that opens opportunities for students to engage in research, work in laboratories, and participate in professional development activities such as conferences. Scholarships that cover high costs such as tuition and living expenses have been critical in increasing participation of PEERs across all BUILD sites. Some institutions such as PSU committed to raising scholarship funds for their first-generation students. The dean explained that such funding helps pay for costs such as tuition. Creating an inclusive environment that is responsive to students’ needs and that ultimately leads to addressing health disparities requires early investments that remove financial barriers.

Financial support also opens students’ networks and chances for gaining opportunities to pursue research in biomedical fields. Some students said that financial barriers limit their participation in professional development experiences. They felt unable to compete with a student who can pay for travel and conference expenses. Addressing these financial barriers can have long-term academic and career benefits for students. For example, the program director at CSUN shared that a BUILD student was specifically looking for a program that offered bioinformatics and found that the leading program was at an out-of-state institution that was not part of CSUN BUILD’s research partner institutions. The program director at CSUN said, "You know what? If this is what you need to get into your doctoral program, then let’s fund you.” The student went on to a summer research program at the out-of-state institution with financial support from BUILD and eventually went on to apply, get accepted, and enroll in the doctoral program at that institution as a result of BUILD’s investment in this summer research opportunity. Funding to complete an internship at an institution outside their partner network helped the student gain experience connected to academic interests and positioned the student to pursue a doctoral program at that institution. Financial support for students’ academic and professional careers results in students’ ability to be more engaged in research and thus more competitive as their career unfolds.

Providing academic support helped students excel in their classes. For some institutions, that meant hiring additional staff to meet students’ needs. For example, CSUN identified writing needs and hired staff that could provide writing support. A CSUN program administrator shared:

We have a very holistic program, I think. We try to do a good [support service] wrap around students, so it’s not just the tutoring, but sometimes it is. Sometimes their writing isn’t strong enough and so I help them with their statements of purpose, but now that the group is so large… right now, we’ve hired two writing coaches. One of them is for academics, so she helps them with their essays and assignments that they have that are writing intensive… she has certain slots that are available just for our students as they need it. We just hired another writing coach that’s focusing on really helping them polish their personal statements and research statements, their statements of purpose to get into graduate study.

CSUN recognized a potential academic barrier and hired staff members to work with students to strengthen writing skills. This helped students better prepare for their coursework, research applications, and have stronger personal and research statements. At other institutions, writing centers were available on campus and the BUILD programs leveraged these existing resources to assist students.

Staff at UMBC were also hired to provide supplemental academic advising to their BUILD students. A program administrator said the academic advisor “isn’t intended to replace their department advising, it’s assigned to supplement their departmental advising.” The supplemental advising was used to help students prepare schedules, prepare for department academic advising appointments, remind students about academic deadlines, and work through academic-related challenges.

How academic support is offered and framed by the institution is also important. Providing academic support due to a deficit view of students is not culturally responsive, but rather can be potentially harmful to students’ science identity development and self-efficacy. However, providing support to reduce barriers for students and begin to change systems to value science for all, rather than science only for those who are academically prepared and high achieving upon college entry, would be an inclusive science approach. A faculty member at CSUN elaborates on the changing mindsets regarding deficit thinking from her faculty peers:

Some faculty, working directly with students they haven’t normally hired or were positioned as those high-achieving students who would normally get the [science training] opportunities… I think some of the faculty have challenged some of their own deficit thinking about the student population here at CSUN in particular. It’s not completely resolved, but it’s started to melt that chilling effect that the science-oriented programs at CSUN had on a lot of the students of color… One of the greatest strengths about the BUILD program [is that it] has been able to bring together faculty… it’s this brain trust across the disciplines that’s really trying to holistically think about how can we work with students of color in the sciences and at the same time, rethink what science means to these students.

Provision of career development opportunities is another form of tangible support that fostered the career development of biomedical students. Career development support came in the form of specific positions created to provide science skill training (e.g., UAF’s Research Advising and Mentoring Professionals), career programs (e.g., workshops to develop CVs and graduate school personal statements), provision of internship and research opportunities, and sending students to biomedical conferences and symposiums. XULA embedded career development into their program model so that career development events and programs were seamlessly integrated into required meetings for BUILD scholars. In partnership with the Student Academic Success Office (SASO), the Center for Undergraduate Research and Graduate Opportunity (CURGO) and the Center for the Advancement of Teaching and Faculty Development (CAT+FD), XULA took the approach of building infrastructure to support additional staff positions in existing offices to bolster career development capacity for BUILD students and all other students majoring in a science discipline.

### Dimension: Partnerships with diverse communities and institutions

An inclusive approach to science includes building and cultivating powerful partnerships with K-12 schools, community colleges, research institutions, and external partners. These partnerships can strengthen participation and address needs to support students’ transitions to and through high school to college to graduate study, and into the workplace. Such partnerships also create additional opportunities for PEER students on their respective campuses. We describe how BUILD sites employed culturally responsive approaches with respect to pipeline partnerships with high schools and community colleges, research institutions, and community partners.

#### Institutionalizing change in recruitment practices through partnerships

Almost all BUILD sites established formalized partnerships with local and regional educational institutions to encourage more PEER students to enter the biomedical sciences at their institution. Stakeholders at many institutions felt that BUILD activities allowed for the prioritization and overall strengthening of relationships with high schools and community colleges (referred to as pipeline partners) with populations of PEER students who can be supported by biomedical training resources at the BUILD site. The partnerships focused on recruitment of PEER students allowed BUILD sites to leverage partnerships that aimed to create lasting change in the compositional diversity of the entering class of first-year or transfer students with biomedical career interests. The culturally responsive recruitment strategies BUILD employed with partner sites also created lasting change in how institutions recruited biomedical talent from their local communities.

Providing students at the pipeline institution with information about how the BUILD site would provide financial support and biomedical research and mentoring opportunities and a more welcoming space for the potential student to thrive was critical in creating successful partnerships.

A community college partner with UDM, for example, articulated how BUILD activities directly strengthened UDM’s relationships with local community colleges:

One of the things that BUILD really was specific in doing was creating that connection…I’d had little interaction with UDM before this. But when I reached out, I had my contact … [who asked], “can you find me the students who’ve been through BUILD before, because there’s a little panel session, and there’s the poster session, and then there’s also a presentation [that is] going to talk about, ‘Here’s how you can come to U of D Mercy. Here’s the benefits, and opportunity.‴ So, really, [BUILD] made that pipeline…

Indeed, experiences from alumni with similar backgrounds to prospective recruits, coupled with the responsiveness of the BUILD site to provide what pipeline partners needed to recruit more students also proved to be helpful in cultivating successful pipeline partners.

Establishing partnerships with local high schools was another recruitment strategy. Several BUILD sites found success establishing partnerships with local high schools. establishing pipeline partnerships can also allow institutions to address local and regional barriers that often bar PEERS from accessing and entering the biomedical sciences. At MSU, two conferences allow for a greater understanding of what BUILD participation can do for students in the local community:

ASCEND [BUILD] does a lot of community outreach and involvement. We have two conferences through the year that center on BUILD. There’s one come September that we outreach to local high schools. But there are, I think, three different high schools and two different middle schools that every year they have this conference they reach out to… about 200 students that didn’t actually attend MSU that were here. And getting them interested, specifically in MSU but also even more so in ASCEND. Getting them in the room and showing them this is the really cool stuff you can do here.

Participants at UTEP described lack of knowledge about biomedical research as one area of disconnect between the university and the local community. At UTEP, BUILD orientation activities serve as a platform to build partnerships and connections with one specific community often ignored by outreach and recruitment efforts: parents. One program administrator explained how targeted outreach to students’ parents helps establish relationships with the community:

We had panelists from our research partners present about what it’s like to try to balance their children, their relationships, and their careers. Those types of workshops, we’re inviting parents as well, are to help parents start to see the possibilities for their students into the future, that they can have families and become research scientists.

UTEP exemplifies how their outreach and recruitment efforts are responsive to the needs and concerns of the individuals in their local community by including parents, guardians, and family members. BUILD sites’ efforts to strengthen the connection between biomedical research training, the institution, and the local population show how biomedical career recruitment efforts can be culturally responsive.

While pipeline partnerships provided opportunities to recruit a more diverse student pool, these intentional connections went beyond providing a stream of potential applicants by also focusing on systemic change. One faculty member at PSU saw the establishment of partnerships with community colleges in the Portland metropolitan area as a way to disrupt the educational impact of gentrification on underrepresented students:

One of the things I think EXITO is really interested in is bringing in [a local community college] because one thing [programs] learned over the last few years is that the gentrification that started in the north Portland area in the mid-80’s has really spread over time to southeast Portland and northeast Portland. And so our students of color are being pushed further and further east…we are able to add that community college to our program and we’re going to spend the next year, then, in the next cohort kind of nurturing [local community college’s] relationship.

Pipeline partnerships also have the ability to change mindsets about which students are able to “succeed” in the biomedical sciences. One faculty participant at a community college explained how the formalized partnership between their institution and CSULB has helped challenge the notion that academic performance as defined by a students’ GPA is the only indicator of the students’ scientific ability:

With the BUILD program, people are starting to realize you can have a student that maybe they’re not the A student of the class, or the student with the 4.0 but they have those [research] skills, and they’ve been able to show them…I’ve seen this in my lab class. I can have a student that has earned a B in my class, but they go to a professional conference with me, and they present their research. And to me, that speaks volumes more about their skill set than their GPA.

In this case from CSULB’s community college partner, the faculty member notes how they are noticing shifts in students’ self-efficacy as future biomedical researchers. Thus, partnerships can create an opportunity for faculty and staff at both the pipeline and receiving institution to influence and track how students are making meaning of their education and career training.

Although BUILD activities helped establish partnerships with local high schools and community colleges, sites like CSULB and UMBC reported that such connections were institutionalized long before BUILD initiatives actualized. One stakeholder at UMBC said that existing partnerships facilitated the efficacy of BUILD activities institutionally, rather than the other way around. Further, though most strove to build (or reinforce currently existing) partnerships with community college, such strides were not universally successful. CSUN struggled to build connections with regional community colleges, with one stakeholder arguing that the presence of larger research institutions in the area led prospective community college transfers to not “want to come to CSUN, [because] they see us as the bad outcome.” Thus, while partnerships are important for recruitment, institutional context may play a role in the success of sustaining or expanding partnerships. In CSUN’s case, the stakeholder alluded to potential competition among several research universities in the same geographic region. Coupled with messaging that students may be receiving from academic advisors about how “competitive, ‘top 100’ STEMM research universities are the main gateways to STEMM doctoral and professional degrees” [[68] p. 115], campuses may need to strategize on what potential partnerships could yield the most synergistic relationships in order to maximize impact. A campus might also commit to systemic change by redoubling efforts to build these partnerships, with an awareness that changing perceptions about an institution’s reputation for successfully fostering biomedical career talent may take time and additional effort.

In short, pipeline partnerships between BUILD institutions and local high schools and community colleges do more than simply alter the compositional diversity of the pool of prospective students for each site. Although not universally successful, pipeline partnerships were one strategy used by many institutions to tailor recruitment and enrollment efforts to address unique demographic challenges, while also addressing broad, systemic challenges facing PEERs in biomedical sciences.

#### Committing to student success through research partnerships

The vast majority of BUILD sites established relationships with colleges, universities, and private research firms to expand student access to research, internships, and other professional development opportunities. Private research partners were particularly receptive to taking BUILD students from several institutions, including XULA and UAF. For some BUILD institutions, private partnerships were perceived as a low-stakes way for research firms to bolster research productivity through an increase in personnel. As one XULA professor noted, “To be honest, the partners, they want our students. They don’t care if they’re BUILD or [another federally funded STEMM scholarship] or not in a program. It’s something that’s fairly low budget, just a little bit of [someone’s coordination] time.” Research partnerships established with other colleges and universities were similarly understood to be mutually beneficial for all parties involved.

Research partnerships allowed for more than just the expansion of available research opportunities; they also provided BUILD sites the ability to address broader, systemic barriers that prevent PEERs from accessing the biomedical sciences. In turn, research partnerships enabled sites such as UAF and SFSU to build stronger connections with local diverse communities. At UAF, partnerships built with researchers in rural Alaska enabled BUILD students to do meaningful work that directly impacted their home communities–labor which research partners described as invaluable. By taking on SFSU students through their BUILD-facilitated research partnership, UCSF similarly was able to expand their own research by directly targeting community-level health disparities.

One SFSU BUILD stakeholder explained how the symbiotic research relationship between their institution and UCSF not only brought research to local communities, but also served to broaden participation of PEERs in biomedical research:

The UCSF folks, they want to work and solve problems in the community, but often they don’t have the access. Whereas our students and students and faculty do have that access, because they are literally from the community. So, it sort of kneads all the things together. [… the project] was very important, not only of course for the communities themselves… but for the students really understanding how what they’re learning on the theoretical level in the classroom is important. I think one thing that comes out in the research over and over, and my own area of expertise in research, is that people, women, and also men of color, are attracted to science and biomedical research when they feel they can give back, and also transform how the science is done. Because they bring a different perspective that hasn’t necessarily been there. That’s something that has been emphasized quite a bit through SF BUILD.

Another way research partnerships helped BUILD sites address systemic barriers to participation in biomedical sciences for underrepresented communities was through shifting the mindsets of external partners on the types of students that can succeed in research internships. Though many institutions formally fostered research partnerships with external stakeholders, such partnerships also formed organically through student and faculty participation in national conferences as supported by BUILD funding. Such was the case for MSU, where one stakeholder described BUILD students as “basically marketing our program” to potential research partners:

They went to the [annual national biomedical conference] a few years ago, and one of our students got an opportunity to intern at Roswell Park Cancer Institute. They were so impressed with her that the director of the program reached out to me and asked if we had any more students like her. He actually came down and spoke to our students during one of our interdisciplinary seminars and had lunch with them [after].

The emphasis BUILD activities place on integrating students into research opportunities and funding student travel to national associations is therefore not only vital to the development of students as biomedical researchers, but also to the development of flagship research partnerships between BUILD institutions and external research entities. Additionally, BUILD activities also carry the powerful potential to shift partners’ understandings about PEER students’ ability to thrive in the biomedical field.

While research partners mostly were receptive to working with BUILD institutions, some sites struggled to establish buy-in from their planned partners. For one BUILD site, the lack of “structured agreements” with research partners adversely impacted buy-in for external institutions to build partnerships: “We don’t have any funds to provide them [… which] has created a lack of continuity with being able to have faculty who are engaged in wanting to be partners with us.” When successfully sustained, however, research partnerships served as a vital strategy for BUILD sites to institutionalize connections with external research entities, expand the availability of research partnerships for students, and, in some cases, work to address structural barriers contributing to underrepresentation in the biomedical sciences.

#### Responding to local needs through community partnerships

BUILD institutions also formed direct partnerships with community organizations, industry partners, and local research centers. BUILD activities facilitated the ability for these organizations to establish trust and contact with the BUILD site. The purposes for establishing community partnerships varied by site and partnership type, ranging from aims to recruit students into a particular biomedical industry to creating new research streams and projects that aimed to solve real-time community problems.

Two BUILD sites–PSU and UDM–described how prior existing relationships with the metropolitan Portland and Detroit areas (respectively) enabled them to build partnerships that served both students and their home communities. PSU, for example, leveraged existing property in Portland to partner with local companies to create physical spaces for students to participate in internships that can better prepare them for industry jobs while working directly in the community: “We can […] hopefully get them so that they get not just the real world experience, but they understand what the real challenges and obstacles that each of these companies sees is important in the future.” Additionally, CSUN was in the middle of organizing a cluster hire for health disparities faculty scholars, with one future long-term goal being that the newly hired faculty would establish community-based participatory research projects that would provide CSUN students with research training experience in addition to support for local community needs. As of 2022, CSUN’s efforts have successfully led to the development of the Health Equity Research and Education Center.

While less common–and sometimes less formalized–than pipeline or research partnerships, partnerships with community programs, companies, and stakeholders were ultimately no less powerful in their ability to help BUILD institutions work towards advancing equity in the biomedical sciences.

### Dimension: Integrating sociali with science identity

Another dimension of Inclusive Science that clearly reflects culturally responsive practice is the intentional integration of participants’ social identities in biomedical training activities and research training. Integrating social identities with science identity involves efforts that support the notion one can be both a scientist and a person with social identities and experiences that are recognized and valued in the biomedical workforce. BUILD sites engaged in the following strategies for helping PEERs integrate social identities with science identity: 1) cultivating identity-based mentorship, 2) exposing students to role models and peers with similar identities, and 3) integrating identities in research and professional development.

Cultivating identity-based mentorship is distinguished by focusing on both providing mentor training and the eventual change in mentoring practices to support students in making connections between the racial and gender identities and their identities as emerging scientists. For example, some campuses focused on increasing biomedical faculty’s culturally aware mentoring skills to foster students’ development of their authentic selves in science. XULA employed a training model called Preparing Mentors and Advisors at Xavier (P-MAX). While many aspects of this training aim to enhance relationships between faculty mentors and mentees engaged in research, topics included in the training include understanding social identities. The training also provides faculty attendees with inclusive mentoring practices that support the multiple identities that students bring into a biomedical research space. Similarly, CSULB adopted a mentor training initiative launched through NRMN called Culturally Aware Mentorship (CAM). The training is aimed at addressing cultural diversity matters in research mentoring relationships. One of the faculty leaders involved in the CAM training shared:

You get your PhD in whatever you get it in, and you’ve had a lot of training in that one area…but we don’t have skill sets in a lot of other areas, and it includes making sure and being vigilant about things like inclusion…We had a large cohort of people [at the mentor training], and we went through and talked very directly about race and white privilege and what this means…you can’t sit there and say, ‘I’m a biologist and everybody’s beautiful on the inside’…Whereas my students don’t have that luxury…We need to be able to talk about it openly.

Having faculty members discuss issues of social identity instead of assuming that biomedical faculty mentors know how to mentor students from diverse backgrounds opens up opportunities for faculty to reflect and grapple with their own assumptions and come up with strategies and tools to enhance mentoring relationships with students who may not share their social identities.

Some campuses took the approach of increasing students’ exposure to scientists who fit one or more of their social identities to increase visibility of biomedical role models who integrate their social identities with their role as scientists. Opportunities to interact with role models provide validation in shared challenges and experiences, as well as mentorship from someone who has already “been there” and whom students may feel more comfortable asking for help. A PSU BUILD program undergraduate student explains how their mentor’s shared Indigenous identity helped create a deeper connection and understanding with respect to navigating science:

My [BUILD program] mentor… she has the same values as me and exercising the importance of taking care of yourself. My career mentor… she’s Native American and I identify with her because my father is an Indigenous Pacific Islander. I care so much about our health too, because after WWII, our diet had changed and everything…I also had a peer mentor before my summer induction and she was great. She is a PSU alumna, she graduated with two majors here [at] PSU, and her master’s degree in education. It was just great because she’s also Pacific Islander and she was the reason why I actually applied [to] PSU and transferred. She helped me with all these financial things I did not understand and encouraged me to pursue my degree outside and just go for it. She also has written letters of recommendation for me and she did so many things to help me transfer…

In this instance, having a mentor with shared identities also coincided with shared values and increased support that the student received from her mentor. Students involved in BUILD also spoke about the value of seeing other peers with intersectional identities (race, gender and scientist identities). A student at CSUN shares how having a community of peers with similar identities and life experiences provides a community of support:

In a Latino family, the woman has to be the one helping out with everything, and I just don’t have time for that anymore. It’s caused so much conflict at home. So, just having other people who understand that, and who are also dealing with and have found ways around it, that’s helpful.

Having access to peer mentors with similar identities and shared experiences helped this BUILD student continue to persist in pursuing a biomedical major and career despite cultural challenges she faced as a Latina woman.

Lastly, sites also aimed to integrate social identities into professional development and research experiences. Prior literature on identity indicates that “one cannot pull off being a particular kind of person (enacting a particular identity) unless one makes visible to (performs for) others one’s competence in relevant practices, and, in response, others recognize one’s performance as credible” [[69] p. 1190]. Thus, based on research of women of color in STEMM, scholars have developed a model for science identity that included three domains: competence, performance, and recognition. Of these, the scholars found that recognition was most important for women of color. Opportunities to integrate identities and obtain culturally relevant professional development can also be found in activities such as the meeting of the Society for the Advancement of Chicanos and Native Americans in Science (SACNAS), the Annual Biomedical Research Conference for Minority Students (ABRCMS), and local science and professional development organizations. Several BUILD sites provided funding and transportation for students to attend these conferences, and students described pivotal and transformative moments that occurred at these annual convenings. These activities allow participants to demonstrate competence in biomedical research activities, perform scientific skills, and obtain recognition from the biomedical community—all the while retaining their cultural and gender identities [69]. Additionally, faculty and students shared how finding connections between biomedical research and one’s social identities or life experiences made research more meaningful for students. A BUILD scholar at PSU describes her undergraduate research experience:

The lab that I’m in now it’s super important that my PI is like, she’s a woman, she’s African-American and we’re working on an African-American community. So it’s like literally all my identity culminated through this project. And then it’s very community-oriented and more social sciences in my first lab and so it really matters to me that it looks like the community, represents the community, and represents me.

This BUILD scholar found representation and meaning in seeing the possibility for alignment between many of her social identities and academic interests. In addition to one-on-one interactions that supported students, some campuses also leveraged their campus contexts as minority serving institutions (MSIs) to support integration of one’s social identities into science identity. Interestingly, the co-authors noticed that because MSIs had high populations of the Black, Latinx, Asian American, or Native American Pacific Islander population that they served, the context of the respective institution offered students a greater sense of belonging and gave space for BUILD to primarily focus on building students’ science identity and preparing them for biomedical careers. For example, MSU (an HBCU) emphasized student ownership of knowledge production and science identity development within the context of a historically black university through their entrepreneurship model of research training, which provides student participants with training to develop research proposals and apply for funding to conduct their studies. A faculty member on the BUILD team at MSU shared a story of a Black student participant’s revelations after starting BUILD at MSU and then going to another state for an external research experience at a predominantly white institution:

I had a conversation with a student just yesterday about his internship… one of the requirements of the program is for the students to do an external internship and we do have pipeline partners that we have worked with… the student mentioned that he was in an apprenticeship model during the summer where he was in a lab doing bench work… The “I can do this,” [mentality] is something that is really important about this [entrepreneurship] program. It does provide them with that sense of self-efficacy. A sense of science identity, you know, that I can come up with this idea and do it…

The student not only shared how much he valued the entrepreneurship model of science training, but how his experience away from MSU also shifted his perspective. The MSU faculty member continued to recount the conversation:

I think that he also felt that there was a need for us to diversify biomedical research. He was at [East coast predominantly white institution for the internship]. He’s an African-American male. There weren’t too many African-American males at the [institution]. It was a different environment than what we have here at a historically black college and university, and so he needed that experience. He needed it, and he benefited from it, but he got a chance to see something a little differently, and he appreciated I think more what we are trying to do here. So, for me, it’s the entrepreneurial model, that component of it, the research that the students are generating themselves; that’s key.

Some sites spoke of the challenges they faced to find faculty who shared similar identities and experiences to the BUILD student participants. Indeed, faculty diversity in STEMM continues to be a critical issue in STEMM education discourse. In these cases, we saw examples from BUILD sites where they provided opportunities for student participants to interact with mentors and role models via conference attendance (e.g., SACNAS) or via professional development organizations. Such strategies can be particularly important to employ for universities that lack faculty diversity.

## Discussion and conclusion

While many may doubt that science can be responsive to diversity and inclusion, the interventions in this study illustrate how colleges and universities can actively engage in culturally responsive practices in STEMM undergraduate research training. The Inclusive Science model expands upon the body of research examining culturally responsive approaches in education [23]. BUILD sites engaged in a variety of practices with intentions to create conditions under which historically excluded groups in biomedical disciplines could achieve success, reduce cultural conflict between students’ lives and their biomedical education and research training, and begin to think critically about the culture of science in relation to their communities [23]. We believe this study contributes an evidence-based framework to the science education literature that can be useful for senior administrators, STEMM program PIs, science faculty, and program staff. These stakeholders are involved in the growing number of STEMM training initiatives on campus and may find this framework’s amalgam of culturally responsive practice literature from education research with actual STEMM education practices from a diverse set of higher education institutions useful. The Inclusive Science model study helps organize and articulate culturally responsive programmatic strategies into a cohesive model that can help higher education organizations think more holistically and structurally about their approach as they develop their own practices to promote inclusive science.

This study utilized evidence to revise the previously conceptualized Inclusive Science model, which was initially developed based on the NIH-funded BUILD initiative’s proposed activities [11]. By analyzing practices from BUILD sites as they implemented their programs to enhance diversity in the biomedical sciences, we documented practices that reveal examples of multiple dimensions of the model. We identified key changes to the model. First, we moved “culturally responsive practice” to the outside of the model after finding evidence that BUILD sites engaged in culturally responsive practices (i.e., centered students’ identity, recognized students’ assets, and addressed larger climate and bias issues that affect PEERs) within all dimensions of Inclusive Science. Additionally, we identified sub-themes within each dimension that further explain culturally responsive strategies that sites employed. Another important expansion of the model was the significant recognition of the need for “Tangible Institutional Support” to categorize how sites provided resources, services, and space to reduce structural and financial barriers to success in biomedical training. In implementing these culturally responsive practices categorized by the Inclusive Science dimensions, institutions began to transform daily practice to prioritize inclusion and student success of PEERs.

This study contributes to the expansion of culturally responsive pedagogy by extending its key principles into STEMM practices that operate inside and outside of the classroom applied within higher education. Culturally responsive practice in biomedical training not only applies to teaching, but to mentoring [55], research training, extra-curricular science involvement, and the additional science socialization that occurs outside of course curriculum.

Together, these domains within the higher education context provide opportunities to support access to skill development, career opportunities, and can influence students’ decisions to pursue biomedical careers. Many of the practices acknowledge systems of oppression in educational and career training, shifting the onus of navigating and persisting in biomedicine toward institutional agents (i.e., faculty, staff, and administrators). Undergirding culturally responsive practices within all the dimensions of Inclusive Science is the need for flexibility in order to best address students’ differing needs. Such practices are responsive in that they actively work toward equity in biomedical training by centering the socialization, experiences, and motivations of individuals from diverse backgrounds in all dimensions of educational and career training. The aim of employing culturally responsive practice is to narrow the opportunity gap through cultivating student academic success, cultural competence, and critical consciousness via daily practices from faculty, staff, and administrators that collectively work to transform systems within STEMM education and training [19].

Another contribution of this study is to provide evidence of culturally responsive strategies that institutions use in their efforts to expand biomedicine to attract and retain students from underrepresented groups. Within each dimension of Inclusive Science, we categorized strategies used by the 10 BUILD sites and highlighted some key examples of culturally responsive approaches. The Inclusive Science model can guide the development of new practices at more institutions that share the same desire to increase participation of diverse researchers in biomedical sciences. The strength of a multiple case study design is that it provides several contexts in which to study the phenomena to generate more insight and nuance that would not have been achieved with only one case [34]. While not all strategies may be possible for a college or university to adopt based on campus context, we have provided examples of a wide range of strategies used by a diverse set of campuses. Institutions interested in advancing equity in science disciplines may find some practical strategies for their own use and adaptation in science training by revising their current curriculum, faculty development, sustaining partnerships, and science training that integrates students’ social and developing science identities.

The Inclusive Science model also contributes to diversity efforts in STEMM by presenting “inclusive science” as a more accurate frame than “inclusive excellence.” Efforts focused on inclusive excellence have neglected culturally responsive practices and often fail to question traditional notions of excellence or relationships with underserved communities. Going beyond inclusive excellence, Inclusive Science aims to provide additional pathways from construction of knowledge, to meaningful partnerships, and addressing specific student needs—recognizing that students arrive with different assets in terms of knowledge, skills and resources. BUILD sites are addressing diversity and inclusion in biomedical training in a way that is unique from what has been done in the past by focusing on equity and systemic, institutional change, rather than solely building a program that can only serve a small group of student scholars. BUILD sites were focused on changing mindsets, physical and institutional structures, and traditional ways of working with communities to rebuild trust in relationships.

Future research can continue to examine culturally responsive strategies that correspond to each dimension of the Inclusive Science model. At the time of site visits, BUILD campuses were beginning to evaluate and measure the extent to which their newly launched initiatives were having an impact on student outcomes. Future research might find other culturally responsive strategies that can be employed in higher education and can be empirically connected to an increase in desired outcomes of retaining PEERs in STEMM. Future studies can link Inclusive Science practices to effectiveness and/or outcomes, such as mapping changes to determine impact or correlations when Inclusive Science practices are implemented.

The impetus for developing these programs to enhance diversity comes from recognizing longstanding disparities among racial/ethnic groups in completion and career achievements in biomedical science [70, 71], as well as from a growing body of studies identifying institutional practices and the effects of systemic racism that produce outcome disparities in science [5]. Although campuses have been engaged in student-centered interventions to diversify the biomedical sciences for over four decades, rising expectations among public and private foundations has resulted in many requesting plans for organizational change and evidence of broader institutionalization of programs and practices that benefit underrepresented groups [72]. Moreover, the racial hate crime and COVID-19 pandemics and their disproportionate effect on minority communities reinforces the need to ensure that these communities are better served in healthcare, involving individuals who are dedicated to allocating the benefits of scientific research in these communities. Now is the time for campuses to learn and focus on developing new strategies to help turn the corner in supporting more student talent from underserved communities for the biomedical workforce.

## Supporting information

S1 Table BUILD sites and institutional characteristics. *Note*: Based on 2020 data from the U.S. Department of Education. AANAPISI = Asian American Native American Pacific Islander-Serving Institution, ANNH = Native Hawaiian-Serving Institution, HBCU = Historically Black College and University, HSI = Hispanic Serving Institution.
(DOCX)
