# Supplementary material for: Enacting inclusive science: Culturally responsive higher education practices in science, technology, engineering, mathematics, and medicine (STEMM)
Source: PLoS One. 2024 Jan 17;19(1):e0293953. doi: 10.1371/journal.pone.0293953 (PMC10793921; doi:10.1371/journal.pone.0293953)
Supplement: S1 Table — Note: Based on 2020 data from the U.S. Department of Education. AANAPISI = Asian American Native American Pacific Islander-Serving Institution, ANNH = Native Hawaiian-Serving Institution, HBCU = Historically Black College and University, HSI = Hispanic Serving Institution. (DOCX) [file pone.0293953.s001.docx]

| Institution | Institutional Type and Minority Serving Institution Status | Institutional Characteristics |
| --- | --- | --- |
| California State University, Northridge (CSUN) | Public  HSI/ AANAPISI | Undergraduate Enrollment: 36,979  Admission Rate: 48%  Pell Grant  recipients: 54% |
| California State University, Long Beach (CSULB) | Public  HSI/AANAPISI | Undergraduate Enrollment: 32,025  Admission Rate: 32%  Pell Grant recipients: 51% |
| Xavier University of Louisiana (XULA) | Private  HBCU | Undergraduate Enrollment: 2,344  Admission Rate: 62%  Pell Grant  recipients: 54% |
| University of Texas at El Paso (UTEP) | Public  HSI | Undergraduate Enrollment: 20,004  Admission Rate: 100%  Pell Grant  recipients: 57% |
| Portland State University (PSU) | Public  Emerging HSI | Undergraduate Enrollment: 19,648  Admission Rate: 89%  Pell Grant  recipients: 40% |
| Morgan State University (MSU) | Public  HBCU | Undergraduate Enrollment: 6,294  Admission Rate: 60%  Pell Grant  recipients: 56% |
| University of Maryland, Baltimore County (UMBC) | Public  AANAPISI | Undergraduate Enrollment: 11,144  Admission Rate: 57%  Pell Grant  recipients: 28% |
| SF BUILD:  BUILD site: San Francisco State University (SF State) & Research-intensive partner institution: University of California, San Francisco (UCSF) | Public // Public  HSI & AANAPISI// N/A | SF State:  Undergraduate Enrollment: 25,867  Admission Rate: 68%  Pell Grant  recipients: 45% |
| ReBUILD Detroit:  BUILD site: University of Detroit Mercy (UDM) & Research-intensive partner institution: Wayne State University (WSU) | Private Catholic  N/A  Public  N/A | Undergraduate Enrollment (UDM): 11,144  Admission Rate (UDM): 78%  Pell Grant  Recipients: 29%  Undergraduate Enrollment (Wayne State): 16,728  Admission Rate (Wayne State): 81%  Pell Grant  recipients: 45% |
| University of Alaska, Fairbanks (UAF) | Public  ANNH | Undergraduate Enrollment: 5,432  Admission Rate: 73%  Pell Grant  recipients: 21% |
